# A comprehensive regional neurochemical theory in depression: a protocol for the systematic review and meta-analysis of 1H-MRS studies in major depressive disorder

**DOI:** 10.1186/s13643-018-0830-6

**Published:** 2018-10-12

**Authors:** Thomas Drago, Patrick W O’Regan, Ivan Welaratne, Shane Rooney, Aoife O’Callaghan, Marissa Malkit, Elena Roman, Kirk J Levins, Lauren Alexander, Denis Barry, Erik O’Hanlon, Veronica O’Keane, Darren William Roddy

**Affiliations:** 1 Trinity College Institute of Neuroscience, Trinity College Dublin, Lloyd Building, Dublin 2, Ireland; 2 Department of Anatomy, Trinity Biomedical Sciences Institute, Trinity College Dublin, Dublin 2, Ireland; 3 Department of Anaesthesia, Intensive Care and Pain Medicine, St. Vincent’s University Hospital, Dublin 4, Ireland; 4 Department of Physiology, School of Medicine, University College Dublin, Dublin 4, Ireland

**Keywords:** Magnetic resonance spectroscopy, Major depressive disorder, Systematic review, Meta-analysis, Metabolite variation

## Abstract

**Background:**

Magnetic resonance spectroscopy (MRS) is a non-invasive analytical technique that investigates the presence and concentrations of brain metabolites. In the context of major depressive disorder (MDD), MRS has revealed regional biochemical changes in GABA, glutamate, and choline across different brain compartments. Technical and methodological advances in MRS data acquisition, in particular proton-based ^1^H-MRS, have resulted in a significant increase in the incidence of reports utilizing the technique for psychiatric disorder research and diagnosis. The most recent comprehensive meta-analysis reviewing MRS in MDD stems from 2006. Using contemporary systemic reviews and meta-analysis, the aim is to first test a neurochemical circuit-based theory of depression and then to determine if clinical scores relate to metabolite concentrations before and during treatment.

**Methods:**

Region-specific metabolite changes in MDD will be assessed by systematic review following the Preferred Reporting Items for Systematic Reviews and Meta-Analyses (PRISMA). Inclusion criteria will include participant age (18 to 65), English language studies, known regions of interest, and detailed documentation of ^1^H-MRS procedures. Reported brain regions will be standardized according neuroanatomical expertise allowing increased power of the meta-analysis. Regions of interest will initially include the hippocampus, thalamus, prefrontal cortex, anterior and posterior cingulate gyri, parietal lobe, and basal ganglia. Exclusion criteria will include comorbid psychiatric illness and drug use. Two independent reviewers will undertake all data extraction, while a third reviewer will check for reviewer discrepancies. Statistical analysis will be performed using STATA supplemented by Metan software and SPSS.

**Discussion:**

This data will shed new light on the biochemical basis of depression in different brain regions, thereby highlighting the potential of MRS in identifying biomarkers and generating models of MDD and treatment response.

**Systematic review registration:**

PROSPERO CRD42018091494

## Background

### Proton magnetic resonance spectroscopy

Magnetic resonance spectroscopy (MRS) is a non-invasive technique that facilitates measurement of biochemical changes in the brain in vivo [1]. It has been demonstrated to provide additional clinically relevant information in a wide variety of conditions, including brain tumors, metabolic conditions, and systemic diseases [2]. The most common form of MRS is ^1^H-MRS or proton MRS. In contrast to other forms of magnetic resonance imaging (MRI) where protons in water molecules generate the overwhelming majority of the signal, ^1^H-MRS analyzes protons attached to molecules other than water. Only small, mobile, highly concentrated molecules (typically > 0.5 μmol/g tissue) can be measured, which in practice limits brain ^1^H-MRS to a restricted number of key metabolites. Useful metabolites including creatine, *N*-acetylaspartate (NAA), myo-inositol, choline, glutamate, glutamine, and gamma-aminobutyric acid (GABA) can be assessed by examining the spectra generated using ^1^H-MRS. Individual metabolite concentrations can be assessed as either absolute concentrations or relative to other molecules (usually creatine) to produce a regionally specific molecular fingerprint. The main limitations of ^1^H-MRS in brain research relate to technicalities in generating robust spectra due to signal to noise ratio concerns. The crude resolution of centimeters compared to millimeters of conventional MRI also limits the precision of the technique in brain research. However, despite these limitations, ^1^H-MRS is becoming increasingly clinically relevant and is also contributing to our understanding of brain diseases such as Parkinson’s disease [3], multiple sclerosis [4], and epilepsy [5]. Similarly, ^1^H-MRS is helping to uncover pathologies in neuropsychiatric illnesses such as schizophrenia [6, 7], bipolar disorder [8, 9], and anxiety [10, 11].

### Major depressive disorder

Major depressive disorder (MDD) is the fourth leading cause of disability worldwide and more than 300 million people suffer from depression globally [12]. Core symptoms of MDD include low mood, anhedonia (loss of pleasure and interest) and sleep, appetite, and energy disturbances [13]. Biological theories of depression include the monoamine hypothesis [14], genetic loading [15], stress response abnormalities [16], neuroplastic changes [17], and neuroinflammation [18]. Neuroimaging has also contributed to the biological understanding of depression. Structural changes in the volumes of brain regions such as the hippocampus [19] and anterior cingulate cortex (ACC) [20] correspond with functional MRI alterations across limbic emotional processing networks [21]. Functional connectivity studies have also shown changes in the connectivity of key limbic areas in depression [22], suggesting that circuit-based abnormalities may underlie some of the symptoms of MDD.

### MRS in major depressive disorder

The most recent fully comprehensive meta-analysis of ^1^H-MRS in MDD, involving multiple brain regions and metabolites, was published in 2006 [23]. Depressed patients had raised basal ganglia choline and decreased frontal Glx when compared to controls. Glx is a compound measure consisting of glutamate, glutamine, and GABA. NAA, myo-inositol, and GABA exhibited no detectable change. Subsequent meta-analyses have concentrated only on specific metabolites or brain regions in MDD. The compound Glx measure was decreased in depression across brain regions in a meta-analysis focused on this metabolite [24]. Specifically, the ACC but not the prefrontal cortex also showed reduced glutamate levels in MDD patients. Similarly, Glx but not glutamate or other metabolites was found to be reduced in a prefrontal cortex-targeted study [25]. A recent meta-analysis of GABA levels across seven psychiatric disorders [26] including MDD showed globally reduced GABA that was state-dependent (i.e., reduced in depression and but not in remission). This supports the GABA-deficit hypothesis of MDD [27]. Moreover, choline has been shown to be elevated in the frontal regions of MDD patients in a recent choline targeted meta-analysis [28]. A number of studies have looked at ^1^H-MRS as a marker of treatment response with antidepressants [29, 30], electroconvulsive therapy [31, 32], transcranial magnetic stimulation [33, 34], and ketamine [35, 36]. To our knowledge, no systematic review or meta-analysis has investigated ^1^H-MRS as a biomarker for treatment response across all metabolites and brain regions.

### Recent advances in ^1^H-MRS

Recent advances in hardware, enhanced automated shimming [37], increasing field strengths [38] (3 T, 4 T, 7 T, and higher), and acquisition protocols [39, 40] allow greater signal to noise ratios than ever before. Corresponding advances in spectral analysis software allow metabolites to be investigated with ever increasing precision. These conditions have resulted in a substantial increase in ^1^H-MRS studies in many psychiatric disorders in recent years. Many of the latest studies in depression target limbic regions such as the hippocampus and subdivisions of the prefrontal and anterior cingulate cortices.

### Objectives and hypothesis

This protocol aims to investigate region-specific metabolite changes in depression through a systematic review following the PRISMA (Preferred Reporting Items for Systematic Reviews and Meta-Analyses) guidelines and subsequent meta-analysis of the data. The protocol is registered with PROSPERO (CRD42018091494). Our primary hypothesis is that changes in metabolite concentrations occur in functionally relevant and integrated brain areas in depression. Our secondary hypothesis is that changes in region-specific metabolite concentrations in depression are related to the severity of depression. This protocol includes meta-regression analysis to examine the relationship between Hamilton Depression Scale (Ham-D) [41] scores and changes in region-specific metabolite changes. Our tertiary hypothesis is that changes in region-specific metabolite concentrations in depression change with treatment. Meta-regression analysis will again be used to examine the relationship between Ham-D changes and metabolite concentrations following treatment. Disproving the null hypothesis may allow us to generate a neurochemical theory of depression across various brain regions.

## Methods

### Search strategy

Online databases including PubMed/MEDLINE, Google Scholar, EMBASE, The Cochrane Library, OVID, and PsycINFO will be systematically examined for articles relating to the proposed hypothesis. To isolate the articles necessary for data extraction, search items will include “DEPRESSION + MRS”, “DEPRESSION + SPECTROSCOPY,” “DEPRESSION + MRI,” “MDD + MRS,” “MDD + SPECTROSCOPY,” “MDD + MRI,” “MOOD DISORDERS + MRS,” “MOOD DISORDERS + SPECTROSCOPY,” “MOOD DISORDERS + MRI,” and “MAGNETIC RESONANCE IMAGING + DEPRESSION” will provide the bulk of the information used in the review. All relevant references in the articles will also be checked and relevant references incorporated into the review. All search items will be re-run just prior to the end publication date to include any new studies.

### Eligibility selection

Studies to be included in the review will focus on ^1^H-MRS studies of patients aged between 18 and 65 with a definite diagnosis of MDD and with comparisons to healthy controls, neither of which possessing a serious comorbid disease. Studies that have no control group will be excluded. There will be clearly defined brain regions of interest (ROI), as well as a clearly documented ^1^H-MRS acquisition protocol. The studies will have clearly documented metabolite concentrations or comparisons. Only peer-reviewed studies will be included. Even though the search terms will be in English, studies returned in all languages will be included. Non-English language studies will be translated using a professional service, and the authors contacted directly if there is any confusion. Exclusion criteria will include evidence of documented illicit drug use in participants, serious comorbid medical illness, significant comorbid psychiatric illness, and studies focused below age 18 and above 65. If data is incomplete or unclear, the first corresponding author and then the final corresponding author will be contacted for the raw data. Conference abstracts and letters will be included only if they contain the sole results from a study and only if they have undergone peer review. Non-peer-reviewed sources, non-proton-based MRS, and unclear metabolite determinations or ratios with no absolute concentrations will also be excluded from the review. If the results of a particular study are reported more than once, the study with the largest sample size will be selected in order to avoid repeated inclusion of data from the same cohorts. Studies reporting measures from more than one anatomical region will be assigned to the dataset as two or more independent rater sets.

### Data collection

Two reviewers will perform all data extraction independently. A third reviewer will check for discrepancies between reviewers and adjust the data accordingly by referring to the original paper. From the extracted data, two further raters will compute the initial effect sizes independently with a third rater reviewing any inconsistencies. Relevant information to be extracted will include the following: (1) authors and year of publication; (2) volumes of interest (VOI) being studied in the article including the size in standardized units and location of the MRS VOI scan; (3) metabolites, absolute and/or relative concentrations, and relative increase or decreases in controls; (4) demographics including number, sex, and age of participants; (5) diagnostic method and HAM-D scores; (6) any treatments, such as antidepressants and duration of treatment, and any other medications being taken along with antidepressants; (7) scanner information, including the model and magnetic field strength (Tesla); and (8) MRS acquisition protocols and spectral processing software.

### Volumes of interest and regions of interest

Many ^1^H-MRS studies use inconsistent or conflicting definitions of brain regions. This can lead to difficulties in inter-study comparisons. As our primary hypothesis aims to inform a neurochemical circuit-based theory of depression, anatomical VOIs will be reclassified into the closest neuroanatomical regions of interest (ROI) as appropriate. Such standardization of regions will allow accurate and reliable comparisons to be drawn between studies. It will also increase the power of the meta-analysis by grouping closely linked structures and allow a systems-based interpretation of results. Initial ROIs will include the hippocampus, thalamus, prefrontal cortex, anterior cingulate, posterior cingulate, parietal lobe, and basal ganglia. VOIs will be reassigned into the most appropriate ROI under the direction of a neuroanatomist. It is expected that some ROIs will need to be reclassified into different ROIs or ROIs may be subdivided as the study progresses.

### Meta-analysis

Statistical analyses will be conducted using STATA (version 15.0 Stata Corp, College Station, TX) supplemented by “Metan” software (Centre for Statistics in Medicine, Oxford, UK). Random effect analysis will be used throughout to weight each study to control for potential heterogeneity [42]. Potential heterogeneity identified in preliminary analysis includes variations in VOI location and volume, within VOI tissue segmentation, echo time, and single-voxel and multi-voxel spectroscopy. Cohen’s *d* statistic will be used for effect sizes and is the difference between the mean of the experimental group and the mean of the comparison group and used as effect sizes. In this study, the mean measure of each metabolite in depressed patients will be subtracted from that in the control group in each ROI respectively and divided by the pooled standard deviation of both. We plan to employ conservative definitions of significance using either Bonferroni [43] or false discovery rate [44] corrections for multiple comparisons as appropriate.

### Sensitivity analysis

We plan to further test the robustness of the findings from the meta-analysis by using sensitivity analysis in ROIs excluding studies with potential confounders. Such confounders may include medication, other medical illnesses, scanner field strength, pulse sequence, and diagnostic scales.

### Meta-regression

Meta-regression analysis will be performed to test our secondary and tertiary hypotheses that neurochemical abnormalities change with depression scores and that metabolite changes occur with treatment. An analysis will be performed to examine the relationship between mean Ham-D score and Cohen’s *d* for metabolite levels in each ROI. For inclusion in meta-regression, the meta-analysis should reveal significant differences between depressed and controls with a sufficient sample size for each ROI to test for meta-regression (*n* > 10) [45]. Similar inclusion criteria will apply for the treatment group. Regression will be performed using SPSS24 (IBM SPSS Statistics 24 for Mac OS), with the level of significance defined by applying Bonferroni or false discovery rate correction as appropriate.

### Between-study heterogeneity

The presence of between-study heterogeneity will be tested using the Cochran Q-statistic, and the magnitude of heterogeneity will be estimated using the *I*^2^ statistic [45]. This measures the proportion of variance of effect size due to heterogeneity. *I*^2^ values of 0.25, 0.50, and 0.75 are considered low, moderate, and high, respectively. A significance level of *p* < 0.10 will be used to establish if studies are heterogeneous. Where a *Q*-statistic is significant, a Galbriath plot will be used to identify those studies that contribute the greatest heterogeneity, allowing us to investigate potential causes [46].

### Bias

Publication bias, the phenomenon where only significant findings get published [47] and small study bias, where smaller studies tend to report larger effect sizes [48] will be examined using Eggers test and funnel plot [49].

### Data synthesis

Demographic, clinical, and methodological variants will be extracted. The total number of participants, studies, ROIs with mean differences, 95% confidence intervals, *p* values, and *I*^2^ statistics will be synthesized in graphical form into a Forest plot [50].

## Conclusions

This is a timely systematic review and meta-analysis of ^1^H-MRS in MDD that is expected to generate insights into the neurochemical basis of depression across anatomically defined brain regions. The objectives of this study are threefold: a comprehensive review of the metabolite change in depression, an investigation of such changes with respect to clinical measures, and finally an investigation of metabolite changes with respect to treatment. A comprehensive study assessing all brain regions and all metabolites may have far-reaching implications for depression research including the identification of disease and treatment biomarkers, thereby enhancing our understanding of the neural pathophysiology of MDD and uncovering potential novel therapeutic targets.

## Abbreviations

ACC: Anterior cingulate cortex
GABA: Gamma-aminobutyric acid
MDD: Major depressive disorder
MRI: Magnetic resonance imaging
MRS: Magnetic resonance spectroscopy
NAA: *N*-Acetylaspartate
PRISMA: Preferred Reporting Items for Systematic Reviews and Meta-Analyses
ROI: Regions of interest
VOI: Volumes of interest
